# Changing Microarthropod Communities in Front of a Receding Glacier in the High Arctic

**DOI:** 10.3390/insects11040226

**Published:** 2020-04-05

**Authors:** Dariusz J. Gwiazdowicz, Bogna Zawieja, Izabella Olejniczak, Piotr Skubała, Anna K. Gdula, Stephen J. Coulson

**Affiliations:** 1 Faculty of Forestry, Poznan University of Life Sciences, Wojska Polskiego 71C, 60-625 Poznań, Poland; annagdula1@gmail.com; 2 Department of Mathematical and Statistical Methods, Poznan University of Life Sciences, Wojska Polskiego 28, 60-637 Poznań, Poland; bogna13@up.poznan.pl; 3 Institute of Biological Sciences, University Cardinal Stefan Wyszynski, Wóycickiego 1/3, 01-938 Warsaw, Poland; iza-olejniczak@wp.pl; 4 Department of Ecology, University of Silesia, Bankowa 9, 40-007 Katowice, Poland; piotr.skubala@us.edu.pl; 5 Swedish Species Information Centre, Swedish University of Agricultural Sciences, ArtDatabanken, Box 7007, 75007 Uppsala, Sweden; stephen.coulson@slu.se; 6 Department of Arctic Biology, University Centre in Svalbard, P.O. Box 156, 9171 Longyearbyen, Norway

**Keywords:** species richness, colonisation, community assembly, dispersal, succession, Spitsbergen

## Abstract

This study was carried out at Ny-Ålesund on Spitsbergen in Svalbard (High Arctic). Eight study sites were established along a transect from the fjord to the snout of the glacier. The sites differed from each other by the type of vegetation cover and soil characteristics. Soil samples were collected and placed in Tullgren funnels. Extracted arthropods were represented by two groups of mites (Mesostigmata and Oribatida) and springtails (Collembola). The pioneer species that occurred first after retreat of the glacier were representatives of the Collembola (*Agrenia bidenticulata* and *Hypogastrura concolor*). Later, other springtails appeared including *Folsomia alpha*, *Folsomia quadrioculata*, *Hypogastrura concolor*, *Isotoma anglicana*, *Sminthurinus concolor* and the first species of oribatid mites; *Camisia foveolata* and *Tectocepheus velatus velatus*. Arthropod communities recorded along the transect were characterized by large variations in both species composition and abundance of individuals. The greater the distance from the glacier snout, the greater the species richness (2 to 22 species). The mean number of species per sample was the lowest at site 8 (1 ± 0.71) (the closest to the glacier) and greatest at site 1 (14 ± 1.41) (furthest from the glacier). The Simpson’s diversity index (D) was distinctly greater at sites 1 (4.61 ± 0.06) and 3 (3.94 ± 0.11) than at other sites, especially site 8 (1.07 ± 0.06). Densities were least in the samples closest to the glacier (30 to 101 individuals; density 3000–10,100 individuals/m2). At the other locations, abundance was highly variable (905 to 7432 individuals; density 90,500–743,200 individuals/m2). The mean abundances were greatest at sites 2 and 3. The great variations in total abundances observed were often due to the presence or absence of one or more dominant species exhibiting extreme abundance variability between sites. The microarthropod community of the High Arctic is composed of heterogeneous circumpolar species, yet on a landscape scale is extremely dependent on local environmental conditions which may be subject to rapid change.

## 1. Introduction

Succession is most simply defined as species change over time [1]. Primary succession is the assembly of ecosystems on barren landscapes following severe disturbances that leave little or no biological legacy (e.g. lava flows, landslides and mine wastes). The assembly process involves colonization of newly exposed substrates and subsequent interactions between the colonizing plants, animals and soil microbes [2].

Community properties, such as species richness and diversity, should change as primary succession progresses [3]. Theory and observations suggest two major patterns of species diversity through succession [4]: diversity can first increase and then decrease asymptotically through succession [5]—this pattern of increasing richness and diversity is very common in early primary succession [6] and has been observed on volcanoes [7] and glacier forelands [8,9]. However, as biomass and cover increase in later succession, competitive dominance can lead to declines in richness and diversity [10]. According to the second pattern, there is a peak in diversity at an intermediate stage of succession, which has been observed in both secondary [4] and primary succession [6]. 

Glacier- forefields provide a unique opportunity to observe the phenomenon of primary succession [11] and several studies on the botanical aspects of glacial retreat exist [12,13]. A prime hypothesis is that the initial nitrogen fixing colonizers determine the initial establishment of late-successional dominants and that other possible causes of successional change need not be invoked [14]. Glacier forelands have been studied extensively with regard to plant succession reviewed by Matthews [6], while the structure of heterotrophic communities during the colonization of deglaciated areas were not recognized until recently [15,16]. The succession of mite species on glacier forelands is also poorly known, as only a few studies have been published [17,18,19]. 

In recent, years a number of taxonomic studies on the arthropod fauna of the Svalbard archipelago have appeared [20]. Several describe new species [21,22], whereas others relate to the revision or redescription of species [23,24]. Studies of faunistic and ecological nature exist [25,26,27], but few papers refer to the differentiation of communities across a glacial foreland [16,18,28,29].

The main aim of this study was to describe the changes occurring alpha and beta diversity after glacial retreat contributing to the understanding of primary succession in post-glacial areas and colonization processes more generally.

## 2. Materials and Methods

### 2.1. Chronosequence

To investigate colonization rates, a consequence approach was employed. Here the arthropod communities are examined along a transect originating at the snout of a retreating glacier and perpendicular to the glacier. Under the assumption that the glacier is retreating then increasing distance from the snout equates to increasing time since release from the ice. Sites laying further from the glacier therefore are older than sites close to the snout and have a longer colonization history.

### 2.2. Study Area

**Figure 1:**
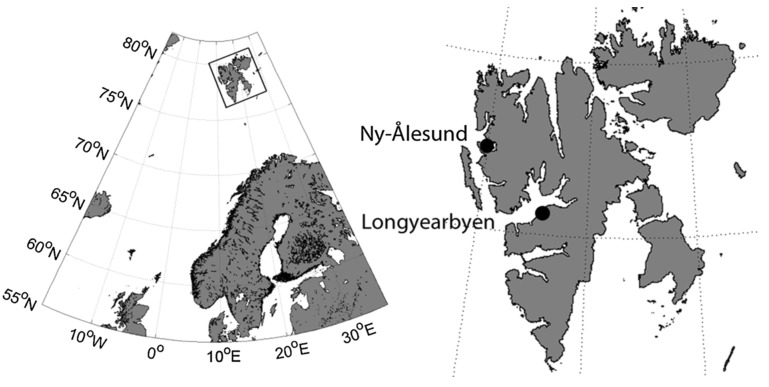
Location of Svalbard (box) and sampling sites at Ny-Ålesund.

The observations were carried out at eight evenly distributed study sites located along a 1.8 km long transect across the glacier foreland of the Midtre Lovén glacier close to Ny-Ålesund (N 78° 53’653′; E 12° 04’797′), on the southern shore of Kongsfjorden, Spitsbergen, Svalbard (Figure 1). The mean monthly air temperature rises above 0 °C for only three months (June to August) and does not exceed 5° [30,31].

To conform to the requirements of the Svalbard Environmental Protection Act of 2001, which aims to minimize damage to sensitive tundra, sampling was restricted to five samples from each location. Each sample consisted of a block of soil turf 10 cm in length and 10 cm in width and extending in depth to the bottom of the organic soil, which ranged between 2 and 4 cm deep. The samples were collected in June 2009. Site one lay furthest from the glacier snout while site 8 was adjacent to the snout. A full consideration of the development of the moss and vascular plant communities is beyond the scope of this article. The most common vascular plant is the pioneer species *Saxifraga oppoistifolia* L.

Site 1—Dense thick layers of moss, grassland turf. >100% vegetation cover. Thick organic soils (>10 cm) (N 78° 54.534′; E 12° 04.537′).

Site 2—Moss-covered surface devoid of other vascular plants (N 78° 54.200′; E 12° 06.763′).

Site 3—Mosses are greater in extent and may have a patch diameter exceeding one meter. Isolated clumps/populations of *S. oppositifolia* L. (N 78° 54.082′; E 12° 06.339′).

Site 4—*S. oppositifolia* occurs along with moss, but they do not form a compact surface. Patch size varies from a few centimeters to greater than one meters (N 78° 53.984′; E 12° 06.085′).

Site 5—Single patches of *S. oppositifolia*. Patches density increasing. The maximum distance between patches is less than four meters (N 78° 53.816′; E 12° 05.590′). No other plants, dry and dusty soil. 

Site 6—Single patches of *S. oppositifolia* spaced between 0.5 to nine meters (N 78° 53.704′; E 12° 05.262′). Patches between 3–5 to 10 × 10 cm. 

Site 7—Single dry fragments of *S. oppositifolia* with a few long branches (N 78° 53.653′; E 12° 04.797′). Distance between plants is several meters.

Site 8—Directly in front of the glacier’s snout on the ice and soil border (N 78° 53.653’; E 12° 04.797’). No plant cover. Muddy, saturated fine silt, gravel and stones. Some ice present.

### 2.3. Laboratory Procedures and Statistics

The soil samples were maintained cool and returned from Ny-Ålesund to the University Centre in Svalbard (UNIS) in Longyearbyen. The samples were placed in Tullgren funnels within 24 hours after collection and extracted for 72 hours until fully dry. The mesofauna was extracted into, and stored, in 96% alcohol. After sorting, slides of the mesostigmatic mites and springtails were prepared using Hoyer’s solution. All oribatid mite measurements were performed on specimens cleared in pure lactic acid and mounted in cavity slides. Identification of oribatid mites to species was based Ghilarov and Krivolutsky [32], Colloff [33], and Weigmann [34]. The classification of Weigmann [34] was followed. Specimens were classified to species under either a light microscope (Zeiss Axioskope 2) or a binocular microscope. All material is deposited in various reference collections at the Department of Arctic Biology, UNIS, Norway, Cardinal Stefan Wyszyński University, Poland (Collembola), Poznan University of Life Sciences, Poland (Mesostigmata), or the University of Silesia, Poland (Oribatida).

To describe the arthropod communities, indices were estimated for each location: diversity; (Simpson’s diversity index (*D*) and Pilou evenness (Evenness index, E = Shannon diversity index /ln [Richness, *S*]). The species abundance was established by determining the number of individuals of each species from each sample.

Statistical approaches widely used in ecological studies, principal coordinates analysis (PCoA), cluster analysis, and indicator species analysis were also performed to explore compositional variation between the samples. Wisconsin double standardization was undertaken before performing the analyses. A linear regression was performed to show biodiversity changes dependent on the distance from the glacier, and hence time, revealed by the Simpson’s index. Cluster analyses were conducted in *STATISTICA*, using the Ward’s method with Manhattan distances. The PCoA, indicator species were calculated using R [35,36,37].

## 3. Results

Forty samples from eight study sites were analyzed. The surfaces closest to the glacier, sites 7 and 8, yielded the fewest individuals (30 to 101; density 3000–10,100 individuals/m^2^) (Table 1). At the other study sites, the densities were very variable (905 to 7,432 individuals; density 90,500–743,200 individuals/m^2^). The mean abundances were greatest at sites 2 (1486.4 ± 2698.6) and 3 (1010 ± 967.1).

The total number of species collected varied from 2 (site 8, close to the glacier snout) to 22 (site 1, farthest away from the glacier) (Table 1). The mean number of species per sample was the least at site 8 (1 ± 0.71) and greatest at site 1 (14 ± 1.41). The Simpson’s diversity indices (*D*) were distinctly greater at sites 1 (4.61 ± 0.08) and 3 (3.94 ± 0.11) than at other sites, especially site 8 (1.07 ± 0.06). *D* for the last site indicates the very small diversity (close to one where one indicates low species diversity). Finally, the evenness indexes (*E*) were the greatest at sites 7 (0.7707) and 1 (0.6277) and the least at sites 8 (0.2108) and 2 (0.2452), while the dominance was greatest at site 8 (0.9356). 

The species composition and structure of the analyzed arthropod communities were different for each study site. From site 8 (in front of the glacier), only two species of springtails were found, *Hypogastrura concolor* (Carpenter, 1900) and *Agrenia bidenticulata* (Tullberg, 1876). At sites 7 and 6, the number of species was greater (7). As the distance from the glacier front increased, the number of species in the samples grew, up to 22 at site 1 (the most distant from the snout of the glacier and the oldest land surface). The community structure also differed across the transect. For example, at site 7 the most numerous species were *Isotoma anglicana* Lubbock, 1862, *H. concolor* and *Folsomia quadrioculata* (Tullberg, 1871), at site 4 the most numerous were *H. concolor*, *Liochthonius laponicus* (Trägårdh, 1910) and *Diapterobates notatus* (Thorell, 1871), while *F. quadrioculata*, *H. concolor* and *Hermannia reticulata* Thorell, 1871 were most dominant at site 1 (Appendix A). 

**Figure 2:**
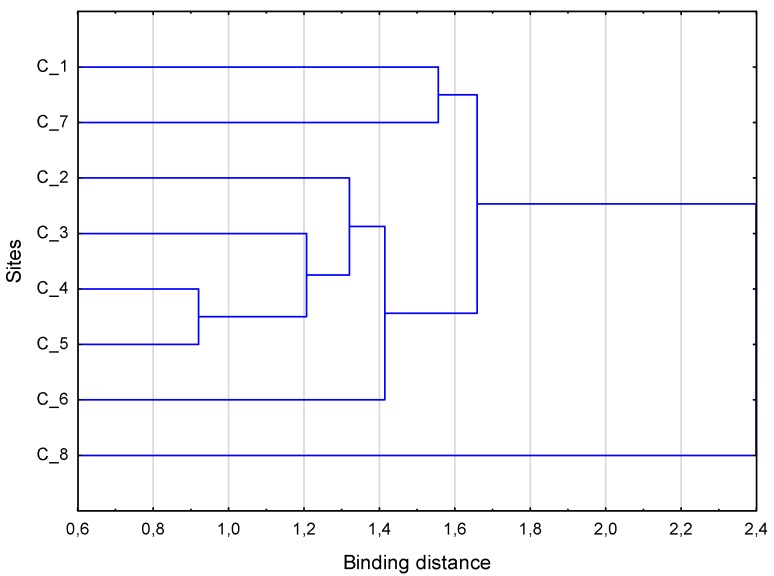
Cluster analysis of microarthropods from the forefield of the Midtre Lovén glacier close to Ny-Ålesund, Svalbard, based on Manhattan dissimilarities of double Wisconsin standardization abundances.

Cluster analysis of pooled samples shows the high similarity of species diversity between sites 4 and 5 as regards occurrence and abundance of species. The sites 3 and 2 are quite similar to 4 and 5, but the outlaying sites are 8, 1 and 7 (Figure 2). Collembola occur at all the sites, while Oribatida do not occur at site 8, and no Mesostigmata occurred at sites 7 and 8. At site 1, there are the greatest number of species. 

**Figure 3:**
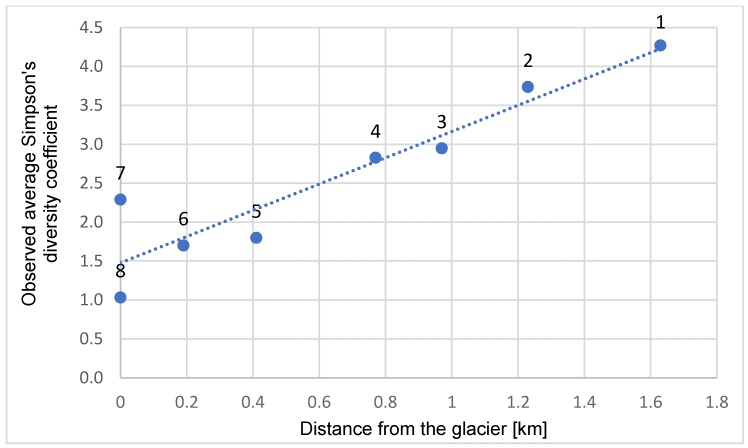
Observed average Simpson’s diversity coefficient and linear regression.

The species richness of the sample grows as the distance from the glacier increases (equivalent to increasing land surface age) (Figure 3), as expressed by both the number of species and the mean Simpson diversity coefficient. The linear regression analysis produced the function *y* = 1.6863^*^ *x* + 1.4779 (^*^ = significance of regression coefficients at < *p* = 0.05, *x* = distance from the glacier [m], *y* = Simpson’s coefficient), with a determination coefficient of *R*^2^ = 68%. Moreover, the Mantel test was used to compare two matrices: Bray Curtis similarities and geographical distances between sites. *r* = 0.174 and *p* = 0.004, indicate that the correlation between these two matrices is significant and that therefore the abundance and occurrence of species depend on distance (site age) from glacier.

**Figure 4:**
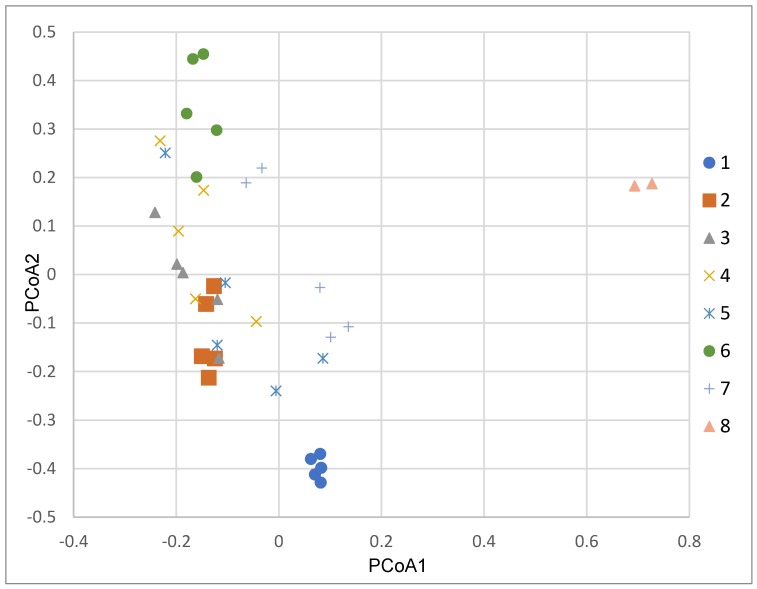
Principal coordinate analysis (PCoA) of sampling units based on Bray–Curtis dissimilarities of double Wisconsin standardization abundances.

Ordination (PCoA) was performed directly on the full (for all samples) dissimilarity matrix (Bray–Curtis on double Wisconsin standardization data) and, although a pattern may be discerned in the differences between assemblages from different sites, there is a great deal of overlap (Figure 4). However, this figure supports the result from the cluster analysis. Sites 1 and 8 are outliers and site 7, although dispersed relative to the vertical axis, is located further to the right of the other sites relative to PCoA1 and sites 4, 5, 3, 2 fall out together. A PERMANOVA analysis (using adonis function) comfirms that effects of sites centroids differ significantly (Table 2). Multivariate dispersions are homogeneous (Permutation test *p* = 0.177), thus the assumption of premutation test is fulfilled. 

The indicator species analysis for pooled samples at the sites identifies 12 species whose groupings distinguish the different sites or groups of sites from each other. Site 1 is distinguished by four species: *Oppiella* (*Moritzoppia*) *neerlandica* (Oudemans, 1900) (only one individual outside site 1), *Hermannia* (*Heterohermannia*) *reticulata* Thörell, 1871 (occurs only at this site), *Arctoseius haarlovi* Lindquist, 1963, and *Oligaphorura ursi* (Fjellberg, 1984)(occurs only in site 1, site 2), *Friesea quinquespinosa* Wahlgren, 1900(only three individual outside site 2), site 3 by *Camisia* (*C*.) *dictyna*, site 5 by *Diapterobates notatus* (Thorell, 1871), and site 8 by *Agrenia bidenticulata* (Tullberg, 1876)(occurs only in this site). The homogenous groups were sites 2 and 3 distinguished by *Arctoseius multidentatus*, 2 and 6 by *Tectocepheus velatus velatus* (Michael, 1880), 3 and 4 by *Liochthonius* (*L*.) *lapponicus*, and 3 and 6 by *Camisia* (*C*.) *foveolata* Hammer, 1955 (Table 3). Although the groups are not the same as in the cluster analysis, the indicator species analysis showed similarities between pairs of sites forming one cluster, additionally indicating the species that are most abundant in a given group.

## 4. Discussion

The areas closest to the glacier were characterized by a low number of species and number of individuals. At site 8 (the closest to the glacier), only 30 individual springtails belonging to two species, *A. bidenticulata* and *H. concolor*, were collected and the latter being represented by only one individual (Appendix A). At site 7, *Folsomia coeruleogrisea*, *F. quadrioculata*, *H. concolor*, *I. anglicana*, and *Sminthurinus concolor* (Meinert, 1896) occurred and two species of oribatid mite, *C. foveolata* and *T. velatus velatus*. These arthropod species can be regarded as pioneer since they first appeared on the early postglacial surfaces. 

*Camisia foveolata* is generally considered as a northern species. It occurs in the boreal region [38] but it is usually not abundant [39]. It is a relatively large mite species, approximately 800 μm in length as an adult. Individuals belonging to the genus *Camisia* secrete an adhesive material onto the cuticle which forms an ornate, transparent cerotegument, to which soil, debris, pollen, spores and fungal hyphae adhere, sometimes aggregated into a thick, compact layer [33]. This cerotegument may aid in camouflage [40]. *Tectocephus velatus* is one of the most frequent and widespread species of oribatid mites occurring throughout the world (including Antarctic and sub-Antarctic islands). This species is without doubt extremely ubiquitous with very wide ecological tolerances and whose habitat can include preserved natural areas or extremely disturbed biotopes, such as agroecosystems [41], urban environments [42], or heavily contaminated post-industrial spoil [43]. It occurs in various moisture regimes from dry steppes to wet grassland and from forests to pioneer stages of succession [44]. Furthermore, it is a parthenogenetic species [45] allowing a rapid population increase after the arrival of a small number of colonizers. *Tectocepheus velatus* is regarded as a panphytophagous animal [46], being an unspecialized feeder on plant material and fungi.

The collembolan *Agrenia bidenticulata* is a bryofile species inhabiting mosses saturated with water and usually occurring at altitudes above 1000 m [47,48]. Individuals have also been found in the litter of an *Aegopodio-Alnetum* forest [49]. *Agrenia* spp. are restricted to colder, wetter regions of the Holarctic and often occur along the margins of small mountain streams under stones [50]. *Hypogastrura concolor* is common around Ny Ålesund [51] and can be found in various tundra habitats especially in moist areas. *Folsomia coeruleogrisea* is an Arctic species common in Svalbard [52]. It prefers open, moist lowland areas, especially meadows or seashores [52,53] and has been recorded from ornithogenic soils in the archipelago [54]. *Folsomia quadrioculata*, a eurytopic species, is widespread throughout the Holarctic [55,56,57,58]. In Arctic regions, *F. quadrioculata* prefers moist areas mainly inhabiting mosses [59]. Another eurytopic species, also avoiding dry sites, is *I. anglicana* found occurring not only in the Arctic regions but also recorded in Central and Northern Europe [52,60]. It is common in Svalbard [61]. *Sminthurinus concolor* is an Arctic species inhabiting various moist tundra habitats especially mosses [53]. *Camisia foveolata* has been so far recorded only from Svalbard [11]. 

The first species of mesostigmatic mites recorded on the post-glacial sites were *Proctolaelaps parvanalis* (Thor, 1930) (site 6), *A. haarlovi* and *A. multidentatus* (site 5). *Proctolaelaps parvanalis* has been reported exclusively from Svalbard [23]. However, it is difficult to conclude if this is an endemic species [27]. On the other hand, both species of *Arctoseius* have been previously recorded in other polar regions, for example in northern Canada or northern Russia [24,62]. The diet of free-living mites of the genus *Arctoseius*, which are numerous in Svalbard, remains unknown. Lindquist considers that they are predators of small arthropods and these conclusions have been confirmed in part by subsequent laboratory studies, for example predating sciarid eggs and first instar larvae [63].

Species richness increased with an increased distance from the glacier. At site 1, the most distant from the glacier, 22 species of microarthropods were recorded. Moreover, this site also displayed the greatest species diversity amongst the eight sites examined. Nine species of Collembola were identified from the foreland of the Midtre Lovén glacier, the most numerous of which were *F. quadrioculata* (582 individuals) and *H. concolor* (315). In addition, *F. quinquespinosa*, *Megaphorura arctica* (Tullberg, 1876) and *O. ursi* were present. These species were not found on the "younger" surfaces closer to the glacier. 

The most numerous species of oribatid mites on this proglacial area were *H. reticulata* (174 individuals) and *Oppiella neerlandica* (134). *Hermannia reticulata* is a species with a boreal distribution [38]. Seyd [64] regards this as a possible relic Arctic ice-age species because it occurs in Britain at a number of high altitude habitats. *Oppiella neerlandica* is characterized by a Holarctic distribution, although it is less common in the southern part of its range [38]. It is most frequently observed in low peat bogs, wet meadows and mosses [34]. It is regarded as a tyrphobiont [65]. The species was associated with the glacier foreland at Nigardsbreen in Norway [19].

The aforementioned species are well adapted to their environment. For example, *M. arctica* and *O. ursi* are typical Arctic species [66] and *F. quadrioculata* can survive on the sea water surface for several days suggesting possible dispersal through the Arctic via oceanic currents [59]. Moreover, due to physiological cryophilic mechanisms, these microarthropods are able to survive at low temperatures for a significant period [67,68,69]; for example, *F. quadrioculata* was shown to be able to survive at −22 °C for four years [70]. They also display phenological adaptations to the Arctic climate, for example *H. concolor*, reproducing in early summer, probably because the optimal conditions for the development of young animals prevail [71] in the spring immediately after snow melt. However, environmental conditions are not the only important factors shaping Collembola communities. The abundance and diversity of these arthropods are greatly influenced by their ability and power of dispersal. The Collembola species identified can all disperse actively and passively, carried by wind, water or by animals where it is especially likely that birds play an important role [72]. Therefore, the number of Collembola species and their occurrence could have been influenced by their high ability to disperse. In addition, their omnivorous life strategy, consuming fungi and bacteria [73] aids colonization.

Site 1 was characterized by more mesostigmatic mites than at the other sites. These included the species *Neoseiulus magnanalis* Thor, 1930 and *Zercon forsslundi* Sellnick, 1958. *Neoseiulus magnanalis* is a species only found on Svalbard in such micro-habitats as, for example, excrement of barnacle goose *Branta leucopsis*, excrement of reindeer *Rangifer tarandus*, and soil of plant communities dominated by polar willow *Salix polaris* [21,74]. *Zercon forsslundi* was recorded in the soil environment in the northern regions of Europe, mainly in Russia, but also in Lithuania and Latvia [75].

## 5. Conclusions

Microarthropod species diversity and abundance increased with increasing distance from the glacier (and time since release from the retreating glacier), mirroring the changes observed in moss and vascular plants diversity and coverage.

Low abundances of microarthropods occurred at the site closest to the glacier snout where vegetation had not become established.

Features of the microarthropod communities (e.g. number of species per sample) were related to site age and plant communities, and varied greatly between transect sites.

Microarthropod species observed were largely generalist circumpolar species, with few endemic or regionally restricted species.

Arctic microarthropod communities can vary greatly across small scale local distances, even when -composed of generalist species with wide circumpolar distributions.

Broadscale generalisations of the distribution of the Arctic microarthropod communities need to take small-grain habitat variation into consideration.

## Figures and Tables

**Table 1:** Diversity of arthropods along a glacier foreland, Midtre Lovén glacier, close to Ny-Ålesund. Data are mean per sample ± standard deviation (SD).

Parameters	1	2	3	4	5	6	7	8
Total number of species	22	16	18	13	11	7	7	2
Mean number of species ± SD	14 ± 1.41	10.6 ± 0.89	10.6 ± 1.82	6.6 ± 0.55	5.4 ± 1.14	4.8 ± 1.30	3 ± 1.00	1 ± 0,71
Total abundance	1521	7432	5050	1067	905	1229	101	30
Mean abundance ± SD	304.2 ± 162.48	1486.4 ± 2698.67	1010 ± 967.12	213.4 ± 139.46	181 ± 199.35	245.8 ± 145.08	20.2 ± 7.95	6 ± 6.67
Simpson (*D*) ± SD	4.61 ± 0.06	1.36 ± 0.29	3.94 ± 0.11	3.46 ± 0.17	2.25 ± 0.19	2.09 ± 0.19	3.43 ± 0.16	1.07 ± 0.05
Evenness (*E*)	0.6277	0.2452	0.5551	0.5950	0.4740	0.4980	0.7707	0.2108
Dominance	0.2170	0.7361	0.2538	0.2893	0.4452	0.4774	0.2916	0.9356

**Table 2:** PERMANOVA analysis of double Wisconsin standardization abundances (1000 permutation).

Effects	Df	SS	MS	Pseudo *F*	*p*
Locations	7	5.9071	0.84386	9.436	0.001
Residuals	31	2.7724	0.08943		
Total	38	8.6795			

**Table 3:** Species that are characteristic of a group of sites revealed using indicator species analysis.

Groups	Species	Stat	*p* Value
1	*Oppiella neerlandica*	0.843	0.001
	*Hermannia reticulata*	0.789	0.001
	*Arctoseius haarlovi*	0.712	0.006
	*Oligaphorura ursi*	0.687	0.008
2	*Friesea quinquespinosa*	0.488	0.044
3	*Camisia dictyna*	0.792	0.001
5	*Diapterobates notatus*	0.676	0.002
8	*Agrenia bidenticulata*	0.785	0.001
2 + 3	*Arctoseius multidentatus*	0.743	0.001
2 + 6	*Tectocepheus velatus*	0.551	0.046
3 + 4	*Liochthonius lapponicus*	0.581	0.026
3 + 6	*Camisia foveolata*	0.627	0.017

## Appendix A

**Table A1:** 1 to 8 in the table legend refer to the sampling sites from the most distant to the closest to the glacier front.

		1			2			3			4			5			6			7			8	
Species	F (%)	A (ind.)	D (%)	F (%)	A (ind.)	D (%)	F (%)	A (ind.)	D (%)	F (%)	A (ind.)	D (%)	F (%)	A (ind.)	D (%)	F (%)	A (ind.)	D (%)	F (%)	A (ind.)	D (%)	F (%)	A (ind.)	D (%)
**Oribatida**																								
*Camisia dictyna* Colloff 1993	80,00	70	4,60	100,0	189	2,54	100,0	1813	35,90	40,00	7	0,66	-	-	-	-	-	-	-	-	-	-	-	-
*Camisia foveolata* Hammer 1955	20,00	1	0,07	60,00	13	0,17	100,0	73	1,45	60,00	9	0,84	20,00	3	0,33	80,00	138	11,23	40,00	6	5,94	-	-	-
*Conchogneta dalecarlica* (Forsslund 1947)	-	-	-	-	-	-	20,00	1	0,02	-	-	-	-	-	-	-	-	-	-	-	-	-	-	-
*Diapterobates notatus* (Thorell 1871)	100,0	91	5,98	100,0	79	1,06	100,0	203	4,02	100,0	133	12,46	100,0	563	62,21	-	-	-	-	-	-	-	-	-
*Hermannia reticulata* Thorell 1871	100,0	174	11,44	-	-	-	-	-	-	-	-	-	-	-	-	-	-	-	-	-	-	-	-	-
*Liochthonius lapponicus* (Trägårdh 1910)	80,00	9	0,59	100,0	40	0,54	100,0	286	5,66	60,00	164	15,37	40,00	15	1,66	80,00	12	0,98	-	-	-	-	-	-
*Liochthonius tuxeni* (Forsslund 1957)	20,00	1	0,07	-	-	-	-	-	-	-	-	-	-	-	-	-	-	-	-	-	-	-	-	-
*Mycobates bicornis* (Strenzke 1954)	-	-	-	20,00	1	0,01	-	-	-	-	-	-	-	-	-	-	-	-	-	-	-	-	-	-
*Mycobates parmeliae* (Michael 1884)	60,00	7	0,46	20,00	4	0,05	-	1	0,02	-	-	-	-	-	-	-	-	-	-	-	-	-	-	-
*Oppiella (Moritzoppia) neerlandica* (Oudemans 1900)	100,0	134	8,81	-	-	-	20,00	1	0,02	-	-	-	-	-	-	-	-	-	-	-	-	-	-	-
*Steganacarus (Atropacarus) striculus* (Koch 1835)	-	-	-	-	-	-	40,00	2	0,04	-	-	-	-	-	-	-	-	-	-	-	-	-	-	-
*Tectocepheus velatus* (Michael 1880)	-	-	-	100,0	485	6,53	100,0	75	1,49	80,00	52	4,87	60,00	79	8,73	100,0	273	22,21	20,00	4	3,96	-	-	-

**Mesostigmata**																								
*Arctoseius haarlovi* Lindquist 1963	100,0	29	1,91	40,00	5	0,07	-	-	-	80,00	32	3,00	60,00	7	0,77	-	-	-	-	-	-	-	-	-
*Arctoseius multidentatus* Evans 1955	80,00	8	0,53	100,0	19	0,26	100,0	27	0,53	20,00	2	0,19	20,00	2	0,22	-	-	-	-	-	-	-	-	-
*Neoseiulus magnanalis* (Thor, 1930)	40,00	19	1,25	40,00	2	0,03	-	-	-	-	-	-	-	-	-	-	-	-	-	-	-	-	-	-
*Proctolaelaps parvanalis* (Thor 1930)	40,00	3	0,20	-	-	-	-	-	-	-	-	-	20,00	2	0,22	20,00	1	0,08	-	-	-	-	-	-
*Zercon forsslundi* Sellnick 1958	20,00	1	0,07	-	-	-	-	-	-	-	-	-	-	-	-	-	-	-	-	-	-	-	-	-
**Collembola**																								
*Agrenia bidenticulata* (Tullberg 1876)	-	-	-	-	-	-	-	-	-	-	-	-	-	-	-	-	-	-	-	-	-	80,00	29	96,67
*Anurida polaris* (Hammer 1954)	-	-	-	20,00	30	0,40	-	-	-	20,00	1	0,09	-	-	-	-	-	-	-	-	-	-	-	-
*Bonetogastrura nivalis* (Martynova 1973)	20,00	3	0,20	-	-	-	-	-	-	-	-	-	-	-	-	-	-	-	-	-	-	-	-	-
*Folsomia coeruleogrisea* (Hammer, 1938)	80,00	19	1,25	-	-	-	-	-	-	-	-	-	20,00	1	0,11	-	-	-	20,00	5	4,95	-	-	-
*Folsomia quadrioculata* (Tullberg 1871)	100,0	582	38,26	100,0	6353	85,48	80,00	1178	23,33	100,0	129	12,09	60,00	26	2,87	60,00	5	0,41	60,00	18	17,82	-	-	-
*Friesea quinquespinosa* Wahlgren 1900	20,00	2	0,13	60,00	19	0,26	20,00	1	0,02	-	-	-	-	-	-	-	-	-	-	-	-	-	-	-
*Hypogastrura concolor* (Carpenter 1900)	100,0	315	20,71	80,00	65	0,87	100,0	1288	25,50	100,0	514	48,17	100,0	201	22,21	100,0	792	64,44	60,00	19	18,81	20,00	1	3,33
*Isotoma anglicana* Lubbock 1862	100,0	24	1,58	80,00	93	1,25	60,00	26	0,51	60,00	19	1,78	40,00	6	0,66	40,00	8	0,65	80,00	47	46,53	-	-	-
*Megaphorura arctica* (Tullberg 1876)	20,00	3	0,20	40,00	35	0,47	-	-	-	-	-	-	-	-	-	-	-	-	-	-	-	-	-	-
*Oligaphorura ursi* (Fjellberg, 1984)	60,00	15	0,99	-	-	-	-	-	-	-	-	-	-	-	-	-	-	-	-	-	-	-	-	-
*Parisotoma notabilis* (Schaeffer 1896)	-	-	-	-	-	-	40,00	68	1,35	-	-	-	-	-	-	-	-	-	-	-	-	-	-	-
*Tetracanthella arctica* Cassagnau 1959	-	-	-	-	-	-	20,00	1	0,02	20,00	1	0,09	-	-	-	-	-	-	-	-	-	-	-	-
*Lepidocyrtus lignorum* (Fabricius 1793)	-	-	-	-	-	-	20,00	5	0,10	-	-	-	-	-	-	-	-	-	-	-	-	-	-	-
*Sminthurinus concolor* (Meinert, 1896)	60,00	11	0,72	-	-	-	20,00	1	0,02	20,00	4	0,37	-	-	-	-	-	-	20,00	2	1,98	-	-	-
Total		1521	100		7432	100		5050	100		1067	100		905	100		1229	100		101	100		30	100
